# Association of Specific Mental Disorders With Premature Mortality in the Danish Population Using Alternative Measurement Methods

**DOI:** 10.1001/jamanetworkopen.2020.6646

**Published:** 2020-06-03

**Authors:** Nanna Weye, Natalie C. Momen, Maria K. Christensen, Kim M. Iburg, Søren Dalsgaard, Thomas M. Laursen, Preben B. Mortensen, Damian F. Santomauro, James G. Scott, Harvey A. Whiteford, John J. McGrath, Oleguer Plana-Ripoll

**Affiliations:** 1 National Centre for Register-Based Research, School of Business and Social Sciences, Aarhus University, Aarhus, Denmark; 2 Department of Public Health, Aarhus University, Aarhus, Denmark; 3 The Lundbeck Foundation Initiative for Integrative Psychiatric Research (iPSYCH), Aarhus, Denmark; 4 Centre for Integrated Register-Based Research, Aarhus University, Aarhus, Denmark; 5 Queensland Centre for Mental Health Research, Brisbane, Queensland, Australia; 6 School of Public Health, University of Queensland, Brisbane, Queensland, Australia; 7 Institute for Health Metrics and Evaluation, University of Washington, Seattle; 8 QIMR Berghofer Medical Research Institute, Herston, Queensland, Australia; 9 Metro North Mental Health Service, Herston, Queensland, Australia; 10 Queensland Centre for Mental Health Research, The Park Centre for Mental Health, Wacol, Queensland, Australia; 11 Queensland Brain Institute, University of Queensland, St Lucia, Queensland, Australia

## Abstract

**Importance:**

The association of mental disorders with premature mortality published in the Global Burden of Disease (GBD) studies has been underestimated because these analyses have recommended using only a small number of mental disorders as causes of death to estimate years of life lost (YLL). Alternative methods have been introduced, such as estimating life-years lost (LYL), to compare individuals with mental disorders with the general population.

**Objectives:**

To generate register-based YLL and LYL estimates and to use these measurement methods to assess the association of specific mental disorders with premature mortality.

**Design, Setting, and Participants:**

This population-based cohort study included all persons with and without mental disorders aged 0 to 94 years who were living in Denmark between January 1, 2000, and December 31, 2015. Data were analyzed from January to December 2019.

**Main Outcomes and Measures:**

Danish health registers were used to identify mental disorder diagnoses, dates of death, and causes of death. The YLLs were estimated for the set of mental health–associated causes of death, and all-cause and cause-specific LYLs were estimated for 18 specific mental disorders and 3 broad categories of mental disorders that were recommended for use in the GBD studies. The association between the number of comorbid mental disorders (divided into categories of persons with ≥1 type of disorder, ≥2 types of disorders, ≥3 types of disorders, and ≥4 types of disorders) and LYL estimates was also examined.

**Results:**

A total of 6 989 627 individuals (3 481 219 male persons [49.8%] and 3 508 408 female persons [50.2%]; mean [SD] age at study enrollment, 32.2 [24.4] years) were followed up for a total of 85 911 461 person-years. The YLL rates per 100 000 person-years were highest for alcohol use disorder (for male individuals, 568.7 [95% CI, 564.4-572.7]; for female individuals, 155.5 [95% CI, 153.5-157.9]) and suicide (for male individuals, 590.1 [95% CI, 583.8-596.5]; for female individuals, 202.3 [95% CI, 198.5-206.4]). Although only 3 of 18 mental and substance use disorders could be associated with YLL, all mental disorders were associated with shorter life expectancies when LYL was used for measurement. Male and female individuals diagnosed with any mental disorder had life expectancies that were shorter by 11.2 years (95% CI, 11.1-11.3 years) and 7.9 years (95% CI, 7.8-8.0 years), respectively, and remaining life expectancy decreased further among those with comorbid mental disorders. Drug use disorders were associated with the highest excess LYL estimates; however, common mental disorders, such as depressive and anxiety disorders, were also associated with substantial premature mortality.

**Conclusions and Relevance:**

Mental disorders were observed to be associated with reductions in life expectancy. This finding provides a foundation for future intervention programs designed to reduce the differential mortality gap associated with mental disorders. Register-based studies allow the calculation of precise individual-level YLLs and LYLs, and both measurement methods are informative for health care planning. Compared with YLL, the novel LYL measurement approach may more precisely capture the association of mental disorders with premature mortality and facilitates the exploration of comorbidity and specific causes of death in individuals with mental disorders.

## Key Points

**Question:**

Are specific types of mental disorders associated with premature mortality that can be quantified using alternative measurement approaches?

**Findings:**

In this population-based cohort study of 6.9 million individuals with and without mental disorders in Denmark, male and female persons diagnosed with any mental disorder had life expectancies of that were 11.2 years and 7.9 years shorter, respectively, compared with the general Danish population. Drug use disorders were associated with the highest rates of excess years of life lost; however, common mental disorders, such as depressive and anxiety disorders, were also associated with substantial premature mortality.

**Meaning:**

The finding that mental disorders are associated with reductions in life expectancy can provide a foundation for future intervention programs designed to reduce the differential mortality gap associated with mental disorders.

## Teaser

This cohort study uses data from Danish health registers to generate estimates of years of life lost and life-years lost and to use these alternative measurement approaches to assess whether specific mental disorders are associated with premature mortality in the Danish population.

## Introduction

Mental disorders are common; approximately one-third of all persons in high-income countries will be treated for a mental disorder at some point in their lives.^1^ In addition, individuals with mental disorders have an increased risk of premature mortality.^2,3^ Estimates of mortality in those with mental disorders are generally presented as mortality rates (ie, the number of deaths among individuals with a disorder per total person-years of observation) or mortality rate ratios (ie, the ratio of mortality rates among those with and without a specific disorder). Another measurement approach that can be used to assess the association between different mental disorders and premature mortality is life expectancy, which is a type of estimate that can be easier to explain to the general public (eg, those with a type of mental disorder die a certain number of years earlier compared with the general population).

A widely used measure of premature mortality associated with life expectancy is years of life lost (YLL), a method used in the landmark Global Burden of Disease (GBD) study.^4^ The calculation of YLL is relatively simple. The difference between the observed age at death and the optimal remaining life expectancy at that age is calculated. The limitations of using YLL to measure excess mortality associated with mental disorders have been debated.^5^ In particular, within the current GBD framework, only 3 causes of death are associated with mental disorders (eating disorders, alcohol use disorders, and drug use disorders); suicide, which is a common cause of death among persons with mental disorders,^6^ is classified as an injury rather than a mental disorder. People with any type of mental disorder are more likely to experience premature mortality, as mental disorders are associated with an increased risk of developing general medical conditions.^7,8,9^ Although excess mortality for many conditions can be ascertained from the GBD data set,^10^ excess mortality associated with mental disorders is not fully captured in the YLLs estimated in the GBD studies. For example, if a person with schizophrenia subsequently develops cardiovascular disease and dies of a myocardial infarction 20 years earlier than the reference population, the entire 20 YLLs for this individual will be categorized as ischemic heart disease and not schizophrenia.

An alternate approach to the estimation of life expectancy is to focus on age at disease onset rather than age at death. Mortality rates for those with a specific disorder can be used to estimate life expectancy for those experiencing the disorder. However, the accurate assessment of premature mortality should consider several factors. Remaining life expectancy varies at each specific age, and traditional measures of disorder-associated life expectancy have made the simplifying assumption that the disorders have a set age of onset (eg, 15 years).^11,12^ In the past decade, methods have been developed that take a more realistic approach to age of onset, namely the estimation of life-years lost (LYL).^13^ After the onset of a disorder (eg, depression) at a specific age, the LYL method estimates the difference in remaining life expectancy compared with the entire population, which is matched by age and sex. A mean LYL estimate for specific disorders can be derived that takes into account all causes of death. Based on this method, life expectancy among those with a mental disorder has been reported as 10.2 years shorter in male individuals and 7.3 years shorter in female individuals compared with the general population.^2^ An additional benefit of this method is that the LYL can be fractionated into specific causes of death.^14^ The LYL estimates associated with broad categories of mental disorders^3^ and schizophrenia^15^ have been published. However, to date, no studies have estimated LYLs in those with a range of specific mental disorders. In particular, LYLs for the mental disorders included in the GBD studies remain to be assessed.

In this study, we estimated both YLLs and LYLs using Danish registers based on individual-level records accounting for the comorbidity between mental disorders. The YLL methodology used in the GBD analyses is based on available country-specific summary data followed by a complex set of adjustments.^16^ However, we were able to estimate the individual-level YLLs that were associated with mental disorders and total them for the entire nation of Denmark; we then compared these data with the summary-level YLLs for Denmark that were reported in the GBD results database.^17^ Based on the same sampling framework, we estimated excess mortality as LYLs that were associated with a comprehensive range of specific mental disorders, and we examined comorbidity within the spectrum of mental disorders and its association with premature mortality.

## Methods

We conducted a population-based cohort study using data from Danish national registers. All citizens and legal residents in Denmark are assigned a unique identification number, which enables linkage between national databases. Using the Danish Civil Registration System,^18^ we identified all persons living in Denmark between January 1, 2000, and December 31, 2015. Persons were included in the study population beginning at the date of birth, the date of immigration to Denmark, or the date on which the study period began (January 1, 2000), whichever occurred last. The follow-up period ended at the date of death, the date of their 95th birthday, the date of emigration from Denmark, or the date on which the study period ended (December 31, 2015), whichever occurred first. All data analyses were performed between January and December 2019. This study followed the Strengthening the Reporting of Observational Studies in Epidemiology (STROBE) reporting guideline for cohort studies. The Danish Data Protection Agency and the Danish Health Data Authority approved this study. Danish law does not require informed consent for register-based studies. All data were deidentified at the individual level.

Diagnoses of all mental disorders were retrieved from the Danish Psychiatric Central Research Register,^19^ which contains information on all admissions to psychiatric inpatient facilities since 1969 and all visits to outpatient psychiatric departments and emergency departments since 1995. The *International Classification of Diseases, Tenth Revision* (*ICD-10*), has been used as the diagnostic system in Danish registers since 1994. The list of mental disorders of interest, which were the same as those included in the 2016 GBD study,^16^ and the *ICD-10* codes and earliest possible age at onset for each disorder are available in eTable 1 in the Supplement. Persons with a diagnosis of a mental disorder between January 1, 1995, and December 31, 1999, were considered cases as of January 1, 2000. The date of onset for incident disorders (ie, those not diagnosed before January 1, 2000) was defined as the date of first contact with a health care facility (inpatient, outpatient, or emergency department visit) after January 1, 2000. Persons diagnosed with multiple mental disorders were considered exposed to each disorder and thus included in multiple disorders concurrently, with each disorder having its own age at onset.

Psychiatric comorbidity was investigated in persons diagnosed with 2 or more mental disorders. We counted the number of psychiatric disorders without assuming any disorder hierarchy and without accounting for the temporal order of separate disorders. Persons with comorbidities were categorized into 3 groups: those diagnosed with 2 or more types of mental disorders, 3 or more types of mental disorders, and 4 or more types of mental disorders. The age at onset was defined as the individual’s age at the date of diagnosis of the second, third, or fourth disorder, respectively.

Dates of death and causes of death were identified based on the underlying cause listed in the Danish Register of Causes of Death.^20^ All causes of death were categorized into 13 nonoverlapping groups: infectious disease, neoplasm, diabetes, disease of the circulatory system, respiratory disease, digestive disease, alcohol use disorder, drug use disorder, eating disorder, suicide, accident, homicide, and other cause (eTable 2 in the Supplement).

### Statistical Analysis

To compare our findings with the YLL methods used in the GBD studies, we calculated YLL for mental disorders that appeared on the GBD-recommended list of causes of death (ie, eating disorder, alcohol use disorder, drug use disorder, and suicide) (eTable 2 in the Supplement). For each person who died of one of these causes, the individual YLL was calculated as the number of years of estimated remaining life expectancy at the age of death (eTable 3 in the Supplement). Age-specific remaining life expectancy was based on the theoretical minimum-risk life table, which is built on the lowest observed mortality rates in populations of more than 5 million people.^21^ Individual-level register-based YLLs were compared with the previously published GBD summary-level YLLs estimated for Denmark.^17^

We estimated LYLs for persons with specific types of mental disorders and persons with 2 or more types of mental disorders. The method and its implementation have previously been described in detail.^2,3,13,22^ In brief, for persons with a specific mental disorder, excess LYLs were quantified as the mean number of years lost after disease diagnosis and before a specific maximum age (set to age 95 years in this study) in excess of that experienced by persons in the general population of the same sex and age. The upper age limit of 95 years, which is greater than the life expectancy of the general Danish population, was set to ensure comparability with previous studies that reported LYL estimates among persons with mental disorders.^2,3^ The excess LYLs were further fractionated into specific causes of death. For less prevalent disorders, we lacked the power to calculate reliable LYL estimates across older age ranges (eg, for individuals with eating disorders, mortality rates after age 70 years were unreliable). To generate more precise LYLs, we adopted a conservative strategy by modeling the survival function after age 50 years or age 70 years, using the observed mortality rates in the general population (details are available in eMethods in the Supplement). Only all-cause LYLs were estimated for these disorders.

The 95% CIs were obtained using a bootstrap analysis with 1000 repetitions. All analyses were performed on the secured platform of Statistics Denmark using R software, version 3.5.2 (R Project for Statistical Computing).

## Results

The study included 6 989 627 individuals (3 481 219 male persons [49.8%] and 3 508 408 female persons [50.2%]) aged 0 to 94 years who were living in Denmark between January 1, 2000, and December 31, 2015. The mean (SD) age at study enrollment was 32.2 (24.4) years. Individuals were followed up for a total of 85 911 461 person-years (42 542 043 person-years for male individuals and 43 369 418 person-years for female individuals) (Table 1). By the end of 2015, 425 927 persons (6.1%) had been diagnosed with at least 1 type of mental disorder; of those, 198 240 individuals (5.7%) were male and 227 732 individuals (6.5%) were female. In addition, 37% of those with mental disorders had 2 or more types of disorders, 12.4% had 3 or more types of disorders, and 4.1% had 4 or more types of disorders. Sex-specific estimates are shown in eTable 4 and eTable 5 in the Supplement.

**Table 1.** Number of Cases, Person-Years at Risk, and Number of Deaths Among Persons With Specific Mental Disorders vs All Persons in Denmark

Variable	Cases, No.	Person-years at risk, No.	Deaths, No. (%)^a^	Rate of death^a^
All persons	6 989 627	85 911 461	833 447 (11.9)	9.7
Persons with specific mental disorders				
Alcohol use disorder	54 910	450 376	16 642 (30.3)	37.0
Drug use disorder	26 771	191 3734	4443 (16.6)	23.2
Opioid use	5764	46 860	1690 (29.3)	36.1
Cannabis use	15 749	102 052	1333 (8.5)	13.1
Cocaine use	1813	11 449	199 (11.0)	17.4
Amphetamine use	2415	16 122	267 (11.1)	16.6
Other	7312	54 666	2097 (28.7)	38.4
Schizophrenia	39 258	368 262	7846 (20.0)	21.3
Bipolar disorder	28 512	214 852	6534 (22.9)	30.4
Depressive disorder	189 475	1 402 090	37 040 (19.5)	26.4
Dysthymia	7029	57 163	1199 (17.1)	21.0
Major depressive disorder	186 243	1 372 623	36 507 (19.6)	26.6
Anxiety disorder	111 980	796 741	7741 (6.9)	9.7
Eating disorder	14 556	117 972	251 (1.7)	2.1
Anorexia	9851	75 388	203 (2.1)	2.7
Bulimia	5595	49 294	76 (1.4)	1.5
Personality disorder	83 747	765 673	8412 (10.0)	11.0
Intellectual disability	22 549	180 617	2427 (10.8)	13.4
Autism spectrum disorder	30 616	193 866	247 (0.8)	1.3
ADHD	46 623	244 380	359 (0.8)	1.5
Conduct disorder	2209	19 681	53 (2.4)	2.7
No. of disorders				
≥1	425 972	3 380 930	64 815 (15.2)	19.2
≥2	157 671	1 140 457	18 903 (12.0)	16.6
≥3	52 657	348 383	6448 (12.2)	18.5
≥4	17 322	105 556	2401 (13.9)	22.7

Abbreviation: ADHD, attention-deficit/hyperactivity disorder.

^a^ The denominators for percentage and rate of death are the number of cases and the person-years at risk for the specific disorder, respectively. Rates are shown per 1000 person-years.

During the follow-up period, 833 447 persons (11.9%) died; of those, 419 959 persons (12.1%) were male and 413 488 persons (11.8%) were female. A total of 21 526 persons (2.6%) died of suicide or of one of the causes included in the GBD list of mental disorder–associated causes of death (Table 2). Suicide accounted for the highest YLL estimates in both sexes, but a marked difference was found in rates per 100 000 person-years by sex (among male individuals, 590.1 [95% CI, 583.8-596.5]; among female individuals, 202.3 [95% CI, 198.5-206.4]). The YLL rates per 100 000 person-years associated with alcohol use disorder were also higher in male individuals (568.7 [95% CI, 564.4-572.7]) than in female individuals (155.5 [95% CI, 153.5-157.9]). With the exception of drug use disorder, register-based YLL rates for 2015 were comparable to the summary-level YLL rates published in the GBD results database^17^ (eg, among male individuals with alcohol use disorder, the YLL rate per 100 000 person-years was 569 in the present study vs 551 [95% CI, 467-655] in the GBD database) (eTable 6 in the Supplement). Absolute YLLs were not comparable across the 2 methods, as the GBD analyses estimated YLLs for a period of 1 year, while we estimated YLLs for a period of 16 years.

**Table 2.** Years of Life Lost Associated With Specific Causes of Death Based on Theoretical Minimum-Risk Life Table

Cause of death	Deaths, No.	Years of life lost (95% CI)	
		Absolute	Rate^a^
Male sex			
Alcohol use disorder	8456	241 937 (240 112-243 622)	568.7 (564.4-572.7)
Drug use disorder	407	16 490 (16 097-16 901)	38.8 (37.8-39.7)
Eating disorder	<3	NS^b^	NS^b^
Suicide	7180	251 047 (248 343-253 747)	590.1 (583.8-596.5)
Female sex			
Alcohol use disorder	2519	67 455 (66 556-68 486)	155.5 (153.5-157.9)
Drug use disorder	181	6257 (5903-6605)	14.4 (13.6-15.2)
Eating disorder	51	2012 (1836-2200)	4.6 (4.2-5.1)
Suicide	2732	87 747 (86 099-89 493)	202.3 (198.5-206.4)

Abbreviation: NS, not shown.

^a^ Rate per 100 000 person-years.

^b^ Estimates based on less than 3 cases are not shown.

With respect to LYLs, all mental disorders were associated with excess LYL estimates compared with the general population (Table 3). The remaining life expectancy after diagnosis of any mental disorder was 11.2 years (95% CI, 11.1-11.3 years) shorter in male individuals and 7.9 years (95% CI, 7.8-8.0 years) shorter in female individuals compared with those in the general population of the same age. The disorder associated with the highest excess LYL estimates was opioid use disorder (for male individuals, 20.1 [95% CI, 19.3-20.9]; for female individuals, 19.0 [95% CI, 18.1-20.0]). Common mental disorders were also associated with substantial premature mortality. For example, in those with major depressive disorders, the excess LYL estimates were 8.3 (95% CI, 8.1-8.5) for male individuals and 6.4 (95% CI, 6.3-6.6) for female individuals. Similar LYL estimates were found in those with anxiety disorders (for male individuals, 7.5 [95% CI, 7.1-7.9]; for female individuals, 6.3 [95% CI, 6.0-6.6]).

**Table 3.** Excess Life-Years Lost Associated With All Causes of Death Among Persons With Any Mental Disorder and Specific Mental Disorders vs the General Danish Population^a^

Mental disorder	Life-years lost (95% CI)		Sex difference (95% CI)
	Male sex	Female sex	
Alcohol use disorder	14.4 (14.2 to 14.7)	13.5 (7.9 to 13.8)	0.9 (0.5 to 1.3)
Drug use disorder	18.0 (17.5 to 18.5)	15.3 (14.7 to 15.9)	2.7 (1.9 to 3.5)
Opioid use	20.1 (19.3 to 20.9)	19.0 (18.1 to 20.0)	1.1 (−0.1 to 2.3)
Cannabis use	15.7 (13.1 to 17.0)	12.2 (8.6 to 16.2)	3.1 (−1.5 to 6.9)
Cocaine use	18.9 (14.2 to 22.3)	15.1 (8.1 to 21.0)	3.8 (−3.7 to 11.4)
Amphetamine use	18.6 (16.2 to 21.0)	13.5 (9.6 to 17.6)	5.2 (0.2 to 9.8)
Other	16.6 (15.8 to 17.4)	12.1 (11.4 to 12.8)	4.5 (3.5 to 5.6)
Schizophrenia	13.8 (13.5 to 14.1)	11.8 (11.4 to 12.1)	2.0 (1.5 to 2.6)
Bipolar disorder	8.8 (8.4 to 9.2)	8.1 (7.7 to 8.4)	0.7 (0.2 to 1.2)
Depressive disorder	8.2 (8.1 to 8.4)	6.4 (6.2 to 6.5)	1.9 (1.6 to 2.1)
Major depressive disorder	8.3 (8.1 to 8.5)	6.4 (6.3 to 6.6)	1.9 (1.6 to 2.1)
Dysthymia	7.5 (6.6 to 8.5)	6.2 (5.5 to 7.0)	1.3 (0.2 to 2.5)
Anxiety disorder	7.5 (7.1 to 7.9)	6.3 (6.0 to 6.6)	1.2 (0.7 to 1.7)
Eating disorder	8.4 (0.8 to 16.1)	7.6 (5.9 to 9.4)	0.7 (−6.6 to 8.4)
Anorexia	9.6 (−2.6 to 18.3)	10.5 (8.4 to 12.6)	−1.2 (−13.2 to 8.4)
Bulimia	6.5 (−3.3 to 17.0)	3.1 (0.4 to 6.7)	3.2 (−6.9 to 14.7)
Personality disorder	10.6 (10.2 to 11.0)	8.5 (8.1 to 8.8)	2.1 (1.6 to 2.7)
Intellectual disability	13.5 (12.9 to 14.1)	14.0 (13.3 to 14.7)	−0.5 (−1.5 to 0.4)
Autism spectrum disorder	8.0 (6.2 to 9.8)	11.3 (7.9 to 13.7)	−3.1 (−6.3 to 0.7)
ADHD	8.0 (5.8 to 10.7)	3.7 (−0.1 to 9.4)	4.7 (−1.7 to 9.2)
Conduct disorder	6.6 (1.8 to 12.3)	10.8 (1.3 to 21.0)	−4.6 (−16.2 to 5.8)
No. of disorders			
≥1	11.2 (11.1 to 11.3)	7.9 (7.8 to 8.0)	3.3 (3.2 to 3.5)
≥2	12.9 (12.7 to 13.2)	9.8 (9.6 to 10.1)	3.1 (2.7 to 3.4)
≥3	15.0 (14.6 to 15.4)	12.3 (11.9 to 12.8)	2.7 (2.1 to 3.3)
≥4	17.4 (16.8 to 18.1)	15.2 (14.3 to 15.9)	2.3 (1.3 to 3.3)

Abbreviation: ADHD, attention-deficit/hyperactivity disorder.

^a^ Persons with any mental disorder and specific mental disorders were compared with persons of the same age and sex in the general Danish population.

Individuals with more than 1 type of mental disorder had an increased number of LYLs, and a dose-response relationship was observed between the number of mental disorders diagnosed and the estimate of LYLs. For example, among men, the LYL estimates were 11.2 (95% CI, 11.1-11.3) for those with one or more type of disorder, 12.9 (95% CI, 12.7-13.2) for those with 2 or more types of disorders, 15.0 (95% CI, 14.6-15.4) for those with 3 or more types of disorders, and 17.4 (95% CI, 16.8-18.1) for those with 4 or more types of disorders (Table 3).

**Figure 1.**
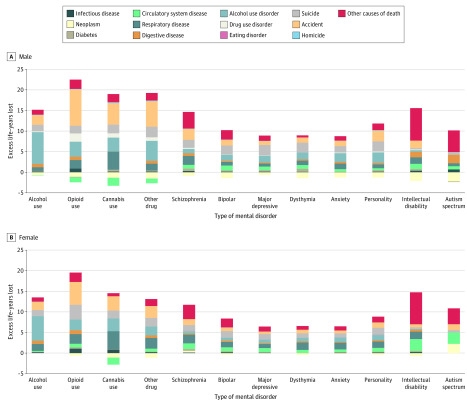
Excess Life-Years Lost Associated With Specific Causes of Death Among Persons With Specific Types of Mental Disorders vs the General Danish Population A, Male. Persons with specific mental disorders were compared with persons of the same age and sex in the general Danish population. Excess life-years lost associated with specific causes of death could not be estimated for disorders with less than 3 cases (cocaine use disorder, amphetamine use disorder, eating disorders, attention-deficit/hyperactivity disorder, and conduct disorder). Estimates and 95% CIs are available in eTable 5 in the Supplement. B, Female. Persons with specific mental disorders were compared with persons of the same age and sex in the general Danish population. Excess life-years lost associated with specific causes of death could not be estimated for disorders with less than 3 cases (cocaine use disorder, amphetamine use disorder, eating disorders, attention-deficit/hyperactivity disorder, and conduct disorder). Estimates and 95% CIs are available in eTable 5 in the Supplement.

**Figure 2.**
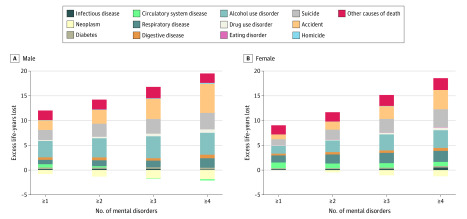
Excess Life-Years Lost Associated With Specific Causes of Death Among Persons With Any Mental Disorder and Multiple Mental Disorders vs the General Danish Population A, Male. Persons with any mental disorder and multiple mental disorders were compared with persons of the same age and sex in the general Danish population. Estimates based on less than 3 cases are not shown. Estimates and 95% CIs are available in eTable 5 in the Supplement. B, Female. Persons with any mental disorder and multiple mental disorders were compared with persons of the same age and sex in the general Danish population. Estimates based on less than 3 cases are not shown. Estimates and 95% CIs are available in eTable 5 in the Supplement.

The excess LYLs for each mental disorder, fractionated into specific causes of death, are shown in Figure 1, Figure 2, and eTable 7 in the Supplement. All mental disorders were associated with excess LYLs compared with the general population for all causes of death, with the exception of neoplasms. In persons with comorbid mental disorders, the additional increased LYLs were associated with accidents, respiratory diseases, alcohol use disorders, and suicide. Those with comorbid disorders had fewer LYLs associated with neoplasms as the cause of death (eg, the LYL estimates among male individuals were −1.3 [95% CI, −1.5 to −1.1] for those with ≥2 types of disorders, −1.7 [95% CI, −2.0 to −1.3] for those with ≥3 types of disorders, and −1.8 [95% CI, −2.5 to −1.1] for those with ≥4 types of disorders) compared with the general population.

## Discussion

To our knowledge, this study is the first to report the association between a broad range of specific mental disorders and comprehensive estimates of premature mortality that were assessed using LYL as a measurement method. We observed that each type of mental disorder was associated with reduced life expectancy compared with the general population. In both female and male individuals, the highest excess LYL estimates were found in individuals with opioid use disorder.

Individuals with any type of mental disorder experienced earlier death compared with the general population, regardless of the method with which premature mortality was measured. Within the set of GBD-recommended mental health–associated causes of death (including suicide), the highest YLL rates were associated with suicide and alcohol use disorders. Eating disorders and drug use disorders were associated with relatively few YLLs.

Our study highlights the ability of register-based studies to calculate precise YLLs based on individual-level observations. Although a substantial body of research exists regarding the burden of infectious diseases estimated using individual-level observations,^23^ this is the first study, to our knowledge, to apply this approach to the analysis of individuals with mental disorders. With the exception of drug use disorders, comparable YLL rates were observed for mental disorders and suicide, as described in the GBD database for 2015. The redistribution of accidental poisonings to the category of drug use disorders in the GBD estimates may explain some of the difference between our estimated YLL rates and those reported in the GBD studies.^24^

With respect to the LYLs that were associated with specific causes of death, estimates are slightly larger than in previous studies of persons with schizophrenia and broad categories of mental disorders.^2,3,15^ However, this is explained by a different time frame and different diagnoses used to define mental disorders (eg, organic disorders were not included in the current study). Consequently, our findings are consistent with previous studies of premature mortality in those with mental disorders. Although suicide accounts for a notable proportion of premature mortality in those with different types of mental disorders, respiratory conditions and alcohol use disorders are also prominent. Of interest, we found that male individuals with a range of mental disorders had a negative estimate of LYLs associated with neoplasms. A previous study reported a similar finding among broader categories of mental disorders,^3^ indicating that (1) male individuals diagnosed with a mental disorder had an increased mortality rate associated with cancer compared with those without a mental disorder but that (2) these individuals had fewer LYLs associated with cancer compared with the general population. Notably, these 2 findings suggest that male individuals with a mental disorder did not live long enough to die of cancer because they were more likely to die of other disorders (eg, diseases of the circulatory system) at a younger age.

While mortality rate ratios have been previously reported for individuals with attention-deficit/hyperactivity disorder, conduct disorder, and autism spectrum disorder,^25,26,27^ this study is, to our knowledge, the first to examine differences in life expectancy for individuals with these disorders. Although nationwide registers were used in this study, the study’s follow-up period for persons diagnosed with mental disorders in childhood or adolescence was too short to accurately estimate LYLs in later adulthood. As a consequence, the 95% CIs for these disorders were wide, and the results should be interpreted with caution. We used a conservative modeling strategy to estimate LYLs in these circumstances; hence, we may have underestimated the LYLs that were associated with these disorders. Despite these limitations, we note that childhood mental disorders were associated with premature mortality, and this association was not detected by the YLL method used in the GBD studies.

To our knowledge, this study provides the first application of the LYL method to measure the association of comorbidity with mental disorders. As expected, individuals diagnosed with 2 or more types of mental disorders experienced a shorter life expectancy compared with those diagnosed with only 1 type of disorder.

### Limitations

This study had several limitations, and our results should be interpreted within the context of those limitations. For each death, we considered only the underlying cause to ensure comparability with the GBD studies; this simplification may have masked the association of other conditions (eg, mental disorders) with premature mortality. Because diagnoses of mental disorders were based on inpatient, outpatient, and emergency department visits, milder cases of mental disorders treated by the general practitioner may have been missed. The use of only hospital visits could have resulted in larger LYL estimates. However, the LYL method likely underestimated the actual LYLs because the first administrative age of disease onset, not the actual age of onset, was used. Although not all register-based diagnoses of mental disorders have been validated, many have been reported to have good results.^28,29,30,31^ In addition, although the study used nationwide health registers and included more than 6.9 million persons, some disorders had too few individuals diagnosed with the conditions to estimate reliable LYLs using observed mortality rates; therefore, a conservative modeling strategy was applied for those disorders. Although our estimates might be reflective of high-income countries in the Nordic region, we cannot assume that our findings can be generalized to countries with different socioeconomic and health care structures.

The YLL and LYL measurement approaches have advantages and disadvantages. The YLL method is simple to use for calculations and provides a framework for making meaningful cross-national comparisons. In addition, YLLs can be estimated even when information on diagnoses is not available, as this method only includes age and cause of death. The YLL approach is also based on discrete types of causes of death; thus, when summed across a population, proportions associated with different types of cause of death can be readily calculated. However, a limited number of mental disorders are currently incorporated in the GBD analyses as potential causes of death to be used in the estimation of YLLs, which may result in an incomplete understanding of overall premature mortality among individuals with mental disorders.^32^ The YLL method only captures the cause of death associated with the single disease with which the death was associated, and the method omits any illnesses that may have had associations with that terminal disease. This omission necessarily results in an underestimation of the YLLs for illnesses associated with mental disorders. In contrast, the LYL method estimates the reduced life expectancy for any disorder or risk factor as a mean for a group of exposed individuals. Because the group is defined as individuals with at least 1 particular type of disorder (eg, those with depression), these individuals may also have a range of other mental and physical disorders, which means that the estimated LYLs inherently account for observed and unobserved comorbidities associated with mortality rates. However, because the LYL measurement is a mean of individual-level mortality, this method is not additive and thus not suitable for analyses that aim to estimate the population burden (ie, analyses similar to those used in the GBD studies).

## Conclusions

The GBD studies have revolutionized our understanding of the burden of disease in populations worldwide. However, mental disorders as a whole are associated with premature mortality, and most of this burden is not detectable by measuring YLLs. To fully quantify the association of mental disorders with the global burden of disease, innovative health measurements for assessing premature mortality, such as LYLs, are needed. Of course, no single mortality-associated health measurement approach can suit all requirements. Better methods are needed to measure the true associations of mental disorders with premature mortality; in particular, these methods are necessary for analysis of disorders that are indirectly associated with excess mortality owing to their associated disability and comorbidity.

## Notes

Supplement. **eMethods.** Life-Years Lost (LYL) Method **eTable 1.** Diagnostic Classification of Mental Disorders Considered in This Study According to *ICD-10* Diagnoses **eTable 2.** Diagnostic Classification of Causes of Death Considered for Years of Life Lost (YLL) Analyses and Life-Years Lost (LYL) Analyses According to *ICD-10* Diagnoses **eTable 3.** Age and Remaining Life Expectancy Estimated Using Theoretical Minimum-Risk Reference Life Tables **eTable 4.** Number of Cases, Person-Years at Risk, and Number of Deaths for All Male Individuals and Male Individuals Diagnosed With Each Specific Mental Disorder **eTable 5.** Number of Cases, Person-Years at Risk, and Number of Deaths for All Female Individuals and Female Individuals Diagnosed With Each Specific Mental Disorder **eTable 6.** Cause-Specific Years of Life Lost (YLL) for Men and Women in Denmark in 2015 **eTable 7.** Cause-Specific Life-Years Lost (LYL) for Each Specific Mental Disorder, Any Mental Disorder, and Multiple Mental Disorders **eReferences.**
