# Supplementary material for: Association of Specific Mental Disorders With Premature Mortality in the Danish Population Using Alternative Measurement Methods
Source: JAMA Netw Open. 2020 Jun 3;3(6):e206646. doi: 10.1001/jamanetworkopen.2020.6646 (PMC7272122; doi:10.1001/jamanetworkopen.2020.6646)
Supplement: Supplement. — eMethods. Life-Years Lost (LYL) Method eTable 1. Diagnostic Classification of Mental Disorders Considered in This Study According to ICD-10 Diagnoses eTable 2. Diagnostic Classification of Causes of Death Considered for Years of Life Lost (YLL) Analyses and Life-Years Lost (LYL) Analyses According to ICD-10 Diagnoses eTable 3. Age and Remaining Life Expectancy Estimated Using Theoretical Minimum-Risk Reference Life Tables eTable 4. Number of Cases, Person-Years at Risk, and Number of Deaths for All Male Individuals and Male Individuals Diagnosed With Each Specific Mental Disorder eTable 5. Number of Cases, Person-Years at Risk, and Number of Deaths for All Female Individuals and Female Individuals Diagnosed With Each Specific Mental Disorder eTable 6. Cause-Specific Years of Life Lost (YLL) for Men and Women in Denmark in 2015 eTable 7. Cause-Specific Life-Years Lost (LYL) for Each Specific Mental Disorder, Any Mental Disorder, and Multiple Mental Disorders eReferences. [file jamanetwopen-3-e206646-s001.pdf]

Supplementary Online Content

Weye N, Momen NC, Christensen MK, et al. Association of specific mental disorders with premature mortality in the Danish population using alternative measurement methods. *JAMA Netw Open.* 2020;3(6):e206646. doi:10.1001/jamanetworkopen.2020.6646

eMethods. Life-Years Lost (LYL) Method

eTable 1. Diagnostic Classification of Mental Disorders Considered in This Study According to *ICD-10* Diagnoses

eTable 2. Diagnostic Classification of Causes of Death Considered for Years of Life Lost (YLL) Analyses and Life-Years Lost (LYL) Analyses According to *ICD-10* Diagnoses

eTable 3. Age and Remaining Life Expectancy Estimated Using Theoretical Minimum-Risk Reference Life Tables

eTable 4. Number of Cases, Person-Years at Risk, and Number of Deaths for All Male Individuals and Male Individuals Diagnosed With Each Specific Mental Disorder

eTable 5. Number of Cases, Person-Years at Risk, and Number of Deaths for All Female Individuals and Female Individuals Diagnosed With Each Specific Mental Disorder

eTable 6. Cause-Specific Years of Life Lost (YLL) for Men and Women in Denmark in 2015

eTable 7. Cause-Specific Life-Years Lost (LYL) for Each Specific Mental Disorder, Any Mental Disorder, and Multiple Mental Disorders

eReferences

This supplementary material has been provided by the authors to give readers additional information about their work.

© 2020 Weye N et al. *JAMA Network Open*.

eMethods. Life-Years Lost Method

The LYL method estimates remaining life expectancy at each possible age of onset. For each age-specific life expectancy, mortality rates from that age until the set upper age limit, in this study set to 95 years, are used. However, if there are no individuals diagnosed and alive in the older ages, the mortality rates for these older ages will be zero, which could underestimate the true LYLs.

We addressed this issue by modelling the mortality rates after certain age thresholds. Disorders that had less than 10 cases near age 70 were estimated using observed mortality rates until age 70 and modelled mortality rates from age 70 years onwards. These disorders were cocaine use disorder, amphetamine use disorder, eating disorders (overall), anorexia, bulimia and ADHD in both men and women. Conduct disorder in men and women had less than 10 cases at age 50 years or earlier and was modelled from age 50 years onwards.

Age-specific life expectancy for each specific mental disorder was estimated using observed mortality rates up until the age threshold. After this age threshold, a conservative approach was applied assuming the mortality rates in the general population to apply in the diseased population.

© 2020 Weye N et al. *JAMA Network Open*.

eTable 1. Diagnostic Classification of Mental Disorders Considered in This Study According to *ICD-10* Diagnoses

**Disorder groups (and subgroups)**	***ICD-10* codes**	**Earliest age of onset (years)**	**Comments**
Alcohol use disorders	F10.2	10	
Drug use disorders (overall)	F11.2, F12.2, F13.2, F14.2, F15.2, F16.2, F18.2	10	
Opioid use disorder	F11.2	10	
Cannabis use disorder	F12.2	10	
Cocaine use disorder	F14.2	10	
Amphetamine use disorder	F15.2	10	
Other drug use disorders	F13.2, F16.2, F18.2	10	
Schizophrenia	F20	10	
Bipolar disorder	F30, F31 (except F31.7), F34.0	10	
Depressive disorders (overall)	F32, F33, F34.1	10	
Major depressive disorder	F32, F33	10	
Dysthymia	F34.1	10	
Anxiety disorders	F40, F41, F42, F43.1, F93.0-F93.2, F93.8	1	
Eating disorders (overall)	F50.0, F50.1, F50.2	1	
Anorexia nervosa	F50.0, F50.1	1	
Bulimia nervosa	F50.2	1	
Personality disorders	F60	10	GBD names this group "Other mental disorders"
Idiopathic developmental intellectual disability	F70-F79, Z81.0	1	
Autism spectrum disorder	F84	1	
ADHD	F90	1	
Conduct disorder	F91.0-F91.2, F91.8	1	
At least one disorder	F10.2, F11.2, F12.2, F13.2, F14.2, F15.2, F16.2, F18.2, F20, F30-F33 (except F31.7), F34.0, F34.1, F40-F42, F43.1, F50.0-F50.2, F60, F70-F79, F84, F90, F91.0-F91.2, F91.8, F93.0-F93.2, F93.8, Z81.0	1	
At least two, three or four disorders	F11.2, F12.2, F13.2, F14.2, F15.2, F16.2, F18.2, F20, F30, F31 (except F31.7), F34.0, F32, F33, F34.1, F40, F41, F42, F43.1, F93.0-F93.2, F93.8, F50, F60, F70-F79.9, Z81.0, F84, F90, F91.0-F91.2, F91.8	1	A person has to be diagnosed with at least two, three or four of the following disorders: alcohol use disorder, amphetamine use disorder, cannabis use disorder, cocaine use disorder, opioid use disorder, other drug use disorders, schizophrenia, bipolar disorder, dysthymia, major depressive disorder, anxiety disorder, anorexia, bulimia, personality disorder, intellectual disability, autism spectrum disorder, ADHD, and conduct disorder.

© 2020 Weye N et al. *JAMA Network Open*.

eTable 2. Diagnostic Classification of Causes of Death Considered for Years of Life Lost (YLL) Analyses and Life-Years Lost (LYL) Analyses According to *ICD-10* Diagnoses

Causes of death	Years of life lost (YLLs)	Life-years lost (LYLs)
Alcohol use disorder	ICD-10: F10-F10.9, G31.2, G72.1, P04.3, Q86.0, R78.0, X45-X45.9, X65-X65.9, Y15-Y15.9	ICD-10: F10, I85, K70
Drug use disorders	ICD-10: F11-F16.9, F18-F19.9, P04.4, P96.1, R78.1-R78.5	ICD-10: F11, F13-F16, F18-F19, P96.1, F78.1-R78.5
Eating disorders	ICD-10: F50.0-F50.5	ICD-10: F50.0-F50.5
Suicide	ICD-10: X60-X64.9, X66-X84.9, Y87.0	ICD-10: X60-X84, Y87.0
Infectious diseases	-	ICD-10: A00-B99
Neoplasms	-	ICD-10: C00-D09
Diabetes mellitus	-	ICD-10: E10-E14
Diseases of the circulatory system	-	ICD-10: F03.9, I00-I25, I27, I30-I52, I60-I84, I86-I99, R54
Respiratory diseases	-	ICD-10: J00-J99
Digestive diseases	-	ICD-10: K00-K69, K71-K93
Accidents	-	ICD-10: V01-X59, Y10-Y86, Y87.2, Y88-Y89
Homicide	-	ICD-10: X85-Y09, Y87.1
Other causes of death	-	The remaining ICD-10 codes or a missing cause of death

© 2020 Weye N et al. *JAMA Network Open*.

eTable 3. Age and Remaining Life Expectancy Estimated Using Theoretical Minimum-Risk Reference Life Tables

Age	Life Expectancy
0	86.6
1	85.8
5	81.8
10	76.8
15	71.9
20	66.9
25	62.0
30	57.0
35	52.1
40	47.2
45	42.4
50	37.6
55	32.9
60	28.3
65	23.8
70	19.4
75	15.3
80	11.5
85	8.2
90	5.5
95	3.7

Theoretical Minimum-Risk Reference Life Tables were the same as those used in the GBD 2016 study.[1] Life expectancy was linearly interpolated within age groups to estimate life expectancy for each year of age. Both columns are in years.

© 2020 Weye N et al. *JAMA Network Open*.

eTable 4. Number of Cases, Person-Years at Risk, and Number of Deaths for All Male Individuals and Male Individuals Diagnosed With Each Specific Mental Disorder

	No. of cases	Person-years at risk	Deaths	Percentage deaths	Rate of deaths
All males living in Denmark	3,481,219	42,542,043	419,959	12.1	9.9
**Specific mental disorders**					
Alcohol use disorder	36,193	291,607	11,430	31.6	39.2
Drug use disorders (overall)	17,613	123,586	2,683	15.2	21.7
Opioid use disorder	3,802	30,617	1,114	29.3	36.4
Cannabis use disorder	11,960	78,339	1,130	9.4	14.4
Cocaine use disorder	1,338	8,136	167	12.5	20.5
Amphetamine use disorder	1,740	11,385	208	12.0	18.3
Other drug disorders	3,309	23,450	905	27.3	38.6
Schizophrenia	22,827	215,428	4,574	20.0	21.2
Bipolar disorder	11,571	84,747	2,722	23.5	32.1
Depressive disorders (overall)	70,042	501,178	14,601	20.8	29.1
Major Depressive Disorder	2,509	19,787	441	17.6	22.3
Dysthymia	68,828	490,607	14,405	20.9	29.4
Anxiety disorders	43,893	300,874	3,117	7.1	10.4
Eating disorders (overall)	690	4,982	15	2.2	3.0
Anorexia	577	4,059	11	1.9	2.7
Bulimia	124	1,013	5	4.0	4.9
Personality disorders	29,542	274,580	3,865	13.1	14.1
Intellectual disability	13,527	108,774	1,303	9.6	12.0
Autism spectrum disorders	22,947	152,064	189	0.8	1.2
ADHD	32,048	180,433	293	0.9	1.6
Conduct disorder	1,691	15,132	38	2.2	2.5
Number of disorders					
1+	198,240	1,540,987	30,666	15.5	19.9
2+	73,177	517,725	9,683	13.2	18.7
3+	24,114	158,244	3,469	14.4	21.9
4+	8,268	50,167	1,333	16.1	26.6

The denominators for percentage and rate of deaths are the number of cases and the person-years at risk for the specific disorder, respectively. Rates are shown per 1,000 person-years.

© 2020 Weye N et al. *JAMA Network Open*.

eTable 5. Number of Cases, Person-Years at Risk, and Number of Deaths for All Female Individuals and Female Individuals Diagnosed With Each Specific Mental Disorder

	No. of cases	Person-years at risk	Deaths	Percentage deaths	Rate of deaths
All females living in Denmark	3,508,408	43,369,418	413,488	11.8	9.5
**Specific mental disorders**					
Alcohol use disorder	18,717	158,770	5,212	27.8	32.8
Drug use disorders (overall)	9,158	67,788	1,760	19.2	26
Opioid use disorder	1,962	16,243	576	29.4	35.5
Cannabis use disorder	3,789	23,713	203	5.4	8.6
Cocaine use disorder	475	3,313	32	6.7	9.7
Amphetamine use disorder	675	4,737	59	8.7	12.5
Other drug disorders	4,003	31,216	1,192	29.8	38.2
Schizophrenia	16,431	152,834	3,272	19.9	21.4
Bipolar disorders	16,941	130,105	3,812	22.5	29.3
Depressive disorders (overall)	119,433	900,912	22,439	18.8	24.9
Dysthymia	4,520	37,377	758	16.8	20.3
Major Depressive Disorder	117,415	882,017	22,102	18.8	25.1
Anxiety disorders	68,087	495,866	4,624	6.8	9.3
Eating disorders (overall)	13,866	112,990	236	1.7	2.1
Anorexia	9,274	71,329	192	2.1	2.7
Bulimia	5,471	48,281	71	1.3	1.5
Personality disorders	54,205	491,093	4,547	8.4	9.3
Intellectual disability	9,022	71,843	1,124	12.5	15.6
Autism spectrum disorders	7,669	41,802	58	0.8	1.4
ADHD	14,575	63,947	66	0.5	1.0
Conduct disorder	518	4,549	15	2.9	3.3
Number of disorders					
1+	227,732	1,839,943	34,149	15.0	18.6
2+	84,494	622,732	9,220	10.9	14.8
3+	28,543	190,139	2,979	10.4	15.7
4+	9,054	55,389	1,068	11.8	19.3

The denominators for percentage and rate of deaths are the number of cases and the person-years at risk for the specific disorder, respectively. Rates are shown per 1,000 person-years.

© 2020 Weye N et al. *JAMA Network Open*.

eTable 6. Cause-Specific Years of Life Lost (YLL) for Men and Women in Denmark in 2015

	GBD 2016	
**Cause of death**	**Absolute YLL (95% CI)**	**Rate of YLL* (95% CI)**
Males		
Alcohol use disorder	15,555 (13,168;18,479)	551 (467;655)
Drug use disorders	7,098 (5,243;8,755)	252 (186;310)
Eating disorders	2 (1;2)	0.1 (0.0;0.1)
Suicide	18,553 (14,741;31,190)	658 (522;1,106)
Females		
Alcohol use disorder	4,308 (3,533;5,147)	151 (124;180)
Drug use disorders	2,036 (1,659;2,488)	71 (58;87)
Eating disorders	69 (51;91)	2 (2;3)
Suicide	5,446 (4,535;6,408)	191 (159;224)

*Rate per 100,000 person-years.

Estimates are based on deaths occurring in 2015. Extracted from GBD Results Tool on 1 November 2018. Downloaded from http://ghdx.healthdata.org/gbd-results-tool on 1 November 2018.

© 2020 Weye N et al. *JAMA Network Open*.

eTable 7. Cause-Specific Life-Years Lost (LYL) for Each Specific Mental Disorder, Any Mental Disorder, and Multiple Mental Disorders

Mental disorder	Cause of death	Excess life-years lost (95% CI)	
		Men	Women
Alcohol use disorder	All cause	14.43 (14.22;14.66)	13.54 (7.94;13.79)
Alcohol use disorder	Infectious diseases	0.20 (0.14;0.27)	0.22 (0.13;0.31)
Alcohol use disorder	Neoplasms	-0.65 (-0.85;-0.45)	-0.02 (-0.3;0.26)
Alcohol use disorder	Diabetes	0.12 (0.04;0.20)	0.11 (0.02;0.20)
Alcohol use disorder	Diseases of the circulatory system	-0.09 (-0.3;0.13)	0.23 (-0.04;0.49)
Alcohol use disorder	Respiratory diseases	0.91 (0.75;1.09)	1.68 (1.45;1.93)
Alcohol use disorder	Digestive disorders	0.79 (0.67;0.90)	0.80 (0.64;0.97)
Alcohol use disorder	Alcohol related	7.72 (7.45;7.98)	5.96 (5.66;6.24)
Alcohol use disorder	Drug related	0.18 (0.14;0.23)	0.17 (0.12;0.22)
Alcohol use disorder	Eating disorders	-	-
Alcohol use disorder	Suicide	1.60 (1.47;1.73)	1.32 (1.17;1.49)
Alcohol use disorder	Accidents	2.35 (2.17;2.51)	1.99 (1.80;2.21)
Alcohol use disorder	Homicide	0.10 (0.07;0.13)	0.05 (0.02;0.08)
Alcohol use disorder	Other causes of death	1.20 (1.03;1.38)	1.03 (0.79;1.26)
Drug use disorders (overall)	All cause	17.99 (17.49;18.53)	15.29 (14.70;15.88)
Drug use disorders (overall)	Infectious diseases	0.46 (0.27;0.66)	0.49 (0.29;0.70)
Drug use disorders (overall)	Neoplasms	-1.18 (-1.67;-0.61)	-1.06 (-1.49;-0.62)
Drug use disorders (overall)	Diabetes	0.25 (0.03;0.48)	0.17 (0.01;0.35)
Drug use disorders (overall)	Diseases of the circulatory system	-0.93 (-1.42;-0.44)	0.52 (0.10;0.98)
Drug use disorders (overall)	Respiratory diseases	2.13 (1.64;2.62)	2.67 (2.21;3.17)
Drug use disorders (overall)	Digestive disorders	0.65 (0.40;0.93)	0.66 (0.41;0.96)
Drug use disorders (overall)	Alcohol related	4.32 (3.80;4.85)	2.70 (2.29;3.14)
Drug use disorders (overall)	Drug related	1.19 (0.97;1.44)	0.70 (0.50;0.93)
Drug use disorders (overall)	Eating disorders	-	-
Drug use disorders (overall)	Suicide	2.38 (2.06;2.75)	2.30 (1.93;2.72)
Drug use disorders (overall)	Accidents	6.29 (5.78;6.81)	3.99 (3.48;4.56)
Drug use disorders (overall)	Homicide	0.24 (0.14;0.34)	-
Drug use disorders (overall)	Other causes of death	2.19 (1.75;2.68)	2.12 (1.66;2.63)
Opioid use disorder	All cause	20.07 (19.32;20.91)	19.00 (18.05;19.98)
Opioid use disorder	Infectious diseases	0.90 (0.54;1.29)	1.09 (0.65;1.64)
Opioid use disorder	Neoplasms	-1.11 (-2.06;-0.21)	-0.63 (-1.54;0.27)
Opioid use disorder	Diabetes	-0.12 (-0.35;0.17)	0.31 (-0.06;0.69)
Opioid use disorder	Diseases of the circulatory system	-1.20 (-1.92;-0.43)	0.88 (0.02;1.85)
Opioid use disorder	Respiratory diseases	2.09 (1.34;3.00)	2.35 (1.48;3.28)
Opioid use disorder	Digestive disorders	0.79 (0.33;1.32)	0.96 (0.46;1.50)
Opioid use disorder	Alcohol related	3.66 (2.80;4.52)	2.58 (1.82;3.32)
Opioid use disorder	Drug related	2.00 (1.54;2.51)	1.24 (0.77;1.75)
Opioid use disorder	Eating disorders	-	-
Opioid use disorder	Suicide	1.86 (1.39;2.37)	2.35 (1.68;3.02)

© 2020 Weye N et al. *JAMA Network Open*.

Opioid use disorder	Accidents	8.78 (7.86;9.68)	5.54 (4.45;6.67)
Opioid use disorder	Homicide	0.15 (0.03;0.30)	-
Opioid use disorder	Other causes of death	2.27 (1.56;3.09)	2.29 (1.39;3.21)
Cannabis use disorder	All cause	15.71 (13.08;17.01)	12.25 (8.60;16.17)
Cannabis use disorder	Infectious diseases	0.16 (-0.04;0.39)	0.84 (0.29;1.47)
Cannabis use disorder	Neoplasms	-1.32 (-2.25;-0.33)	-1.08 (-2.69;0.75)
Cannabis use disorder	Diabetes	0.49 (-0.05;1.27)	0.09 (-0.31;0.64)
Cannabis use disorder	Diseases of the circulatory system	-1.95 (-2.61;-1.13)	-1.72 (-2.67;-0.59)
Cannabis use disorder	Respiratory diseases	4.36 (2.01;5.53)	4.40 (1.68;7.89)
Cannabis use disorder	Digestive disorders	0.01 (-0.23;0.26)	-
Cannabis use disorder	Alcohol related	3.42 (2.65;4.17)	3.11 (1.69;4.76)
Cannabis use disorder	Drug related	1.04 (0.74;1.38)	0.35 (0.06;0.77)
Cannabis use disorder	Eating disorders	-	-
Cannabis use disorder	Suicide	2.10 (1.64;2.58)	1.58 (0.69;2.61)
Cannabis use disorder	Accidents	5.16 (4.42;5.90)	3.47 (2.39;4.66)
Cannabis use disorder	Homicide	0.24 (0.10;0.39)	-
Cannabis use disorder	Other causes of death	1.99 (1.20;2.88)	0.75 (-0.41;2.1)
Other drug disorders	All cause	16.60 (15.80;17.39)	12.09 (11.44;12.76)
Other drug disorders	Infectious diseases	0.17 (-0.06;0.43)	0.24 (0.07;0.43)
Other drug disorders	Neoplasms	-1.52 (-2.20;-0.78)	-1.10 (-1.55;-0.55)
Other drug disorders	Diabetes	0.25 (-0.07;0.64)	0.13 (-0.03;0.33)
Other drug disorders	Diseases of the circulatory system	-1.13 (-1.82;-0.36)	0.72 (0.21;1.21)
Other drug disorders	Respiratory diseases	1.67 (1.05;2.29)	2.60 (2.12;3.14)
Other drug disorders	Digestive disorders	0.68 (0.31;1.10)	0.61 (0.33;0.89)
Other drug disorders	Alcohol related	4.87 (4.07;5.72)	2.17 (1.75;2.60)
Other drug disorders	Drug related	0.85 (0.53;1.18)	0.46 (0.25;0.68)
Other drug disorders	Eating disorders	-	-
Other drug disorders	Suicide	2.60 (2.00;3.23)	1.64 (1.28;2.03)
Other drug disorders	Accidents	6.15 (5.29;7.02)	2.88 (2.32;3.34)
Other drug disorders	Homicide	0.18 (0.05;0.35)	-
Other drug disorders	Other causes of death	1.84 (1.11;2.57)	1.69 (1.22;2.17)
Schizophrenia	All cause	13.80 (13.47;14.14)	11.77 (11.38;12.13)
Schizophrenia	Infectious diseases	0.33 (0.20;0.46)	0.16 (0.07;0.27)
Schizophrenia	Neoplasms	-0.85 (-1.19;-0.52)	0.42 (0.07;0.77)
Schizophrenia	Diabetes	0.56 (0.39;0.73)	0.43 (0.28;0.57)
Schizophrenia	Diseases of the circulatory system	0.93 (0.57;1.29)	1.39 (1.06;1.72)
Schizophrenia	Respiratory diseases	2.15 (1.84;2.44)	2.12 (1.83;2.41)
Schizophrenia	Digestive disorders	0.63 (0.45;0.81)	0.25 (0.10;0.41)
Schizophrenia	Alcohol related	1.07 (0.85;1.27)	0.58 (0.42;0.74)
Schizophrenia	Drug related	0.29 (0.21;0.38)	0.09 (0.03;0.14)
Schizophrenia	Eating disorders	-	0.03 (0.01;0.07)
Schizophrenia	Suicide	1.94 (1.70;2.18)	1.39 (1.17;1.61)
Schizophrenia	Accidents	2.63 (2.37;2.89)	1.44 (1.20;1.67)
Schizophrenia	Homicide	0.07 (0.03;0.12)	-

© 2020 Weye N et al. *JAMA Network Open*.

Schizophrenia	Other causes of death	4.04 (3.70;4.42)	3.48 (3.10;3.87)
Bipolar disorder	All cause	8.81 (8.40;9.17)	8.06 (7.73;8.40)
Bipolar disorder	Infectious diseases	0.19 (0.08;0.30)	0.22 (0.13;0.32)
Bipolar disorder	Neoplasms	-1.43 (-1.76;-1.08)	-0.36 (-0.64;-0.04)
Bipolar disorder	Diabetes	0.25 (0.09;0.42)	0.24 (0.13;0.34)
Bipolar disorder	Diseases of the circulatory system	1.19 (0.79;1.56)	0.97 (0.71;1.23)
Bipolar disorder	Respiratory diseases	0.90 (0.63;1.18)	1.29 (1.05;1.53)
Bipolar disorder	Digestive disorders	0.28 (0.12;0.46)	0.33 (0.20;0.47)
Bipolar disorder	Alcohol related	1.47 (1.18;1.78)	0.72 (0.57;0.89)
Bipolar disorder	Drug related	0.06 (0.01;0.12)	0.07 (0.03;0.13)
Bipolar disorder	Eating disorders	-	-
Bipolar disorder	Suicide	2.17 (1.88;2.46)	1.55 (1.31;1.78)
Bipolar disorder	Accidents	1.38 (1.13;1.64)	0.83 (0.66;1.02)
Bipolar disorder	Homicide	0.04 (0.00;0.10)	0.05 (0.01;0.09)
Bipolar disorder	Other causes of death	2.30 (1.95;2.69)	2.15 (1.88;2.42)
Depressive disorders (overall)	All cause	8.25 (8.07;8.43)	6.38 (6.23;6.53)
Depressive disorders (overall)	Infectious diseases	0.15 (0.10;0.20)	0.10 (0.07;0.14)
Depressive disorders (overall)	Neoplasms	-0.65 (-0.81;-0.49)	-0.08 (-0.20;0.06)
Depressive disorders (overall)	Diabetes	0.37 (0.29;0.45)	0.14 (0.09;0.18)
Depressive disorders (overall)	Diseases of the circulatory system	0.90 (0.74;1.08)	0.99 (0.88;1.11)
Depressive disorders (overall)	Respiratory diseases	0.72 (0.60;0.84)	1.01 (0.91;1.11)
Depressive disorders (overall)	Digestive disorders	0.24 (0.17;0.31)	0.24 (0.18;0.30)
Depressive disorders (overall)	Alcohol related	1.68 (1.53;1.81)	0.85 (0.77;0.94)
Depressive disorders (overall)	Drug related	0.07 (0.04;0.10)	0.04 (0.03;0.06)
Depressive disorders (overall)	Eating disorders	-	0.01 (0.00;0.01)
Depressive disorders (overall)	Suicide	2.53 (2.38;2.67)	1.25 (1.16;1.35)
Depressive disorders (overall)	Accidents	0.93 (0.83;1.04)	0.60 (0.54;0.68)
Depressive disorders (overall)	Homicide	0.03 (0.01;0.05)	0.02 (0.01;0.03)
Depressive disorders (overall)	Other causes of death	1.28 (1.13;1.43)	1.21 (1.11;1.32)
Major Depressive Disorder	All cause	8.27 (8.10;8.47)	6.40 (6.25;6.55)
Major Depressive Disorder	Infectious diseases	0.15 (0.10;0.20)	0.11 (0.07;0.14)
Major Depressive Disorder	Neoplasms	-0.65 (-0.81;-0.47)	-0.08 (-0.22;0.06)
Major Depressive Disorder	Diabetes	0.37 (0.29;0.45)	0.14 (0.09;0.19)
Major Depressive Disorder	Diseases of the circulatory system	0.90 (0.73;1.08)	1.00 (0.88;1.11)
Major Depressive Disorder	Respiratory diseases	0.71 (0.59;0.83)	0.99 (0.88;1.10)
Major Depressive Disorder	Digestive disorders	0.24 (0.17;0.32)	0.24 (0.18;0.29)
Major Depressive Disorder	Alcohol related	1.67 (1.54;1.81)	0.85 (0.77;0.93)

© 2020 Weye N et al. *JAMA Network Open*.

Major Depressive Disorder	Drug related	0.07 (0.04;0.10)	0.04 (0.03;0.06)
Major Depressive Disorder	Eating disorders	-	0.01 (0.00;0.01)
Major Depressive Disorder	Suicide	2.56 (2.40;2.72)	1.27 (1.18;1.35)
Major Depressive Disorder	Accidents	0.92 (0.81;1.03)	0.60 (0.54;0.67)
Major Depressive Disorder	Homicide	0.03 (0.01;0.05)	0.02 (0.00;0.03)
Major Depressive Disorder	Other causes of death	1.30 (1.14;1.45)	1.22 (1.11;1.33)
Dysthymia	All cause	7.54 (6.62;8.52)	6.25 (5.46;7.01)
Dysthymia	Infectious diseases	0.14 (-0.13;0.44)	-0.01 (-0.13;0.13)
Dysthymia	Neoplasms	-1.45 (-2.32;-0.51)	-0.42 (-1.08;0.23)
Dysthymia	Diabetes	0.70 (0.25;1.27)	0.07 (-0.10;0.28)
Dysthymia	Diseases of the circulatory system	0.93 (-0.03;1.90)	0.72 (0.15;1.30)
Dysthymia	Respiratory diseases	1.08 (0.40;1.80)	1.81 (1.22;2.37)
Dysthymia	Digestive disorders	0.27 (-0.10;0.71)	0.26 (-0.01;0.61)
Dysthymia	Alcohol related	1.67 (0.98;2.49)	0.87 (0.50;1.27)
Dysthymia	Drug related	-	-
Dysthymia	Eating disorders	-	-
Dysthymia	Suicide	2.42 (1.64;3.18)	1.08 (0.71;1.48)
Dysthymia	Accidents	1.26 (0.68;1.94)	0.89 (0.51;1.26)
Dysthymia	Homicide	-	-
Dysthymia	Other causes of death	0.50 (-0.16;1.28)	0.89 (0.41;1.41)
Anxiety disorders	All cause	7.52 (7.13;7.92)	6.32 (6.03;6.61)
Anxiety disorders	Infectious diseases	0.08 (-0.02;0.19)	0.11 (0.04;0.18)
Anxiety disorders	Neoplasms	-1.25 (-1.62;-0.88)	-0.20 (-0.48;0.06)
Anxiety disorders	Diabetes	0.08 (-0.06;0.24)	0.15 (0.06;0.25)
Anxiety disorders	Diseases of the circulatory system	0.59 (0.21;0.99)	0.65 (0.41;0.90)
Anxiety disorders	Respiratory diseases	1.37 (1.06;1.68)	1.63 (1.39;1.88)
Anxiety disorders	Digestive disorders	0.40 (0.22;0.57)	0.21 (0.10;0.32)
Anxiety disorders	Alcohol related	2.13 (1.85;2.41)	1.08 (0.93;1.23)
Anxiety disorders	Drug related	0.15 (0.09;0.22)	0.05 (0.02;0.08)
Anxiety disorders	Eating disorders	-	0.01 (0.00;0.03)
Anxiety disorders	Suicide	1.57 (1.34;1.78)	0.93 (0.81;1.07)
Anxiety disorders	Accidents	1.29 (1.07;1.50)	0.70 (0.58;0.83)
Anxiety disorders	Homicide	0.04 (0.00;0.09)	0.00 (-0.01;0.02)
Anxiety disorders	Other causes of death	1.08 (0.77;1.40)	1.01 (0.79;1.23)
Personality disorders	All cause	10.61 (10.20;11.03)	8.47 (8.12;8.78)
Personality disorders	Infectious diseases	0.16 (0.05;0.28)	0.16 (0.08;0.25)
Personality disorders	Neoplasms	-1.23 (-1.60;-0.86)	-0.40 (-0.68;-0.12)
Personality disorders	Diabetes	0.36 (0.20;0.54)	0.20 (0.10;0.30)
Personality disorders	Diseases of the circulatory system	0.48 (0.06;0.87)	0.92 (0.64;1.20)
Personality disorders	Respiratory diseases	0.92 (0.64;1.23)	1.54 (1.29;1.78)
Personality disorders	Digestive disorders	0.34 (0.19;0.52)	0.32 (0.19;0.45)
Personality disorders	Alcohol related	2.57 (2.28;2.88)	1.26 (1.10;1.43)
Personality disorders	Drug related	0.19 (0.12;0.26)	0.12 (0.08;0.17)
Personality disorders	Eating disorders	-	0.03 (0.01;0.06)

© 2020 Weye N et al. *JAMA Network Open*.

Personality disorders	Suicide	2.49 (2.23;2.75)	1.62 (1.46;1.79)
Personality disorders	Accidents	2.59 (2.32;2.86)	1.27 (1.11;1.44)
Personality disorders	Homicide	0.12 (0.07;0.18)	0.02 (0.00;0.05)
Personality disorders	Other causes of death	1.62 (1.27;1.95)	1.40 (1.16;1.65)
Intellectual disability	All cause	13.52 (12.89;14.13)	14.05 (13.32;14.74)
Intellectual disability	Infectious diseases	0.38 (0.17;0.63)	0.26 (0.07;0.49)
Intellectual disability	Neoplasms	-2.09 (-2.58;-1.51)	-0.72 (-1.29;-0.07)
Intellectual disability	Diabetes	0.38 (0.11;0.69)	0.29 (0.07;0.53)
Intellectual disability	Diseases of the circulatory system	1.30 (0.68;1.97)	2.88 (2.16;3.55)
Intellectual disability	Respiratory diseases	1.59 (1.11;2.07)	1.79 (1.27;2.37)
Intellectual disability	Digestive disorders	1.31 (0.89;1.74)	0.49 (0.21;0.81)
Intellectual disability	Alcohol related	0.42 (0.07;0.78)	0.15 (-0.07;0.39)
Intellectual disability	Drug related	0.15 (0.03;0.30)	-
Intellectual disability	Eating disorders	-	-
Intellectual disability	Suicide	0.34 (0.08;0.65)	0.41 (0.18;0.68)
Intellectual disability	Accidents	1.83 (1.34;2.36)	0.74 (0.42;1.08)
Intellectual disability	Homicide	-	-
Intellectual disability	Other causes of death	7.88 (7.05;8.71)	7.76 (6.86;8.69)
Autism spectrum disorders	All cause	8.03 (6.22;9.83)	11.29 (7.86;13.71)
Autism spectrum disorders	Infectious diseases	0.71 (-0.21;2.14)	-
Autism spectrum disorders	Neoplasms	-2.15 (-3.83;-0.03)	2.26 (-1.64;7.00)
Autism spectrum disorders	Diabetes	-	-
Autism spectrum disorders	Diseases of the circulatory system	0.77 (-1.49;3.02)	2.76 (-1.01;7.25)
Autism spectrum disorders	Respiratory diseases	0.72 (-0.71;2.50)	-
Autism spectrum disorders	Digestive disorders	2.06 (0.69;3.68)	-
Autism spectrum disorders	Alcohol related	-0.10 (-0.81;0.81)	-
Autism spectrum disorders	Drug related	-	-
Autism spectrum disorders	Eating disorders	-	-
Autism spectrum disorders	Suicide	0.66 (0.10;1.26)	0.57 (-0.12;1.58)
Autism spectrum disorders	Accidents	-0.11 (-0.58;0.48)	1.44 (-0.19;4.07)
Autism spectrum disorders	Homicide	-	-
Autism spectrum disorders	Other causes of death	5.25 (2.79;7.89)	3.85 (0.28;7.82)
1+	All cause	11.22 (11.09;11.35)	7.89 (7.76;8.01)
1+	Infectious diseases	0.20 (0.16;0.24)	0.14 (0.11;0.17)
1+	Neoplasms	-0.81 (-0.92;-0.68)	-0.02 (-0.13;0.09)
1+	Diabetes	0.31 (0.26;0.36)	0.18 (0.14;0.22)
1+	Diseases of the circulatory system	0.67 (0.55;0.79)	1.01 (0.92;1.12)
1+	Respiratory diseases	0.89 (0.81;0.98)	1.25 (1.17;1.34)
1+	Digestive disorders	0.50 (0.43;0.56)	0.31 (0.26;0.36)
1+	Alcohol related	3.31 (3.19;3.43)	1.38 (1.30;1.45)
1+	Drug related	0.17 (0.14;0.20)	0.06 (0.05;0.08)
1+	Eating disorders	-	0.03 (0.02;0.03)
1+	Suicide	2.06 (1.96;2.15)	1.09 (1.02;1.15)
1+	Accidents	1.92 (1.81;2.02)	0.83 (0.77;0.89)

© 2020 Weye N et al. *JAMA Network Open*.

1+	Homicide	0.07 (0.05;0.09)	0.02 (0.01;0.03)
1+	Other causes of death	1.93 (1.82;2.06)	1.61 (1.52;1.70)
2+	All cause	12.91 (12.68;13.16)	9.81 (9.60;10.05)
2+	Infectious diseases	0.24 (0.17;0.31)	0.18 (0.12;0.25)
2+	Neoplasms	-1.32 (-1.53;-1.12)	-0.41 (-0.61;-0.21)
2+	Diabetes	0.27 (0.17;0.37)	0.16 (0.09;0.22)
2+	Diseases of the circulatory system	0.28 (0.05;0.51)	0.82 (0.63;0.99)
2+	Respiratory diseases	1.20 (1.03;1.38)	1.60 (1.44;1.78)
2+	Digestive disorders	0.57 (0.47;0.68)	0.42 (0.33;0.52)
2+	Alcohol related	3.87 (3.64;4.09)	2.06 (1.91;2.22)
2+	Drug related	0.29 (0.23;0.35)	0.13 (0.09;0.16)
2+	Eating disorders	-	0.04 (0.02;0.06)
2+	Suicide	2.65 (2.47;2.83)	1.74 (1.61;1.88)
2+	Accidents	2.79 (2.61;2.97)	1.41 (1.28;1.54)
2+	Homicide	0.10 (0.06;0.13)	0.02 (0.01;0.04)
2+	Other causes of death	1.99 (1.77;2.20)	1.64 (1.47;1.81)
3+	All cause	15.02 (14.56;15.44)	12.34 (11.89;12.79)
3+	Infectious diseases	0.25 (0.11;0.39)	0.23 (0.12;0.35)
3+	Neoplasms	-1.70 (-2.04;-1.33)	-0.93 (-1.30;-0.60)
3+	Diabetes	0.31 (0.14;0.51)	0.21 (0.08;0.34)
3+	Diseases of the circulatory system	-0.08 (-0.49;0.33)	0.79 (0.46;1.14)
3+	Respiratory diseases	1.35 (1.01;1.68)	1.82 (1.51;2.15)
3+	Digestive disorders	0.49 (0.30;0.68)	0.43 (0.26;0.61)
3+	Alcohol related	4.43 (4.04;4.85)	2.83 (2.52;3.12)
3+	Drug related	0.52 (0.40;0.67)	0.29 (0.19;0.38)
3+	Eating disorders	-	0.04 (0.00;0.07)
3+	Suicide	2.97 (2.65;3.30)	2.41 (2.15;2.69)
3+	Accidents	4.06 (3.67;4.42)	2.24 (1.97;2.53)
3+	Homicide	0.13 (0.06;0.21)	0.06 (0.01;0.11)
3+	Other causes of death	2.29 (1.90;2.66)	1.93 (1.61;2.28)
4+	All cause	17.40 (16.75;18.06)	15.16 (14.30;15.94)
4+	Infectious diseases	0.10 (-0.08;0.33)	0.44 (0.19;0.72)
4+	Neoplasms	-1.84 (-2.50;-1.14)	-1.10 (-1.69;-0.48)
4+	Diabetes	0.38 (0.02;0.75)	0.27 (0.05;0.52)
4+	Diseases of the circulatory system	-0.27 (-1.01;0.51)	0.73 (0.08;1.32)
4+	Respiratory diseases	1.92 (1.26;2.60)	1.95 (1.39;2.57)
4+	Digestive disorders	0.71 (0.34;1.10)	0.52 (0.17;0.86)
4+	Alcohol related	4.42 (3.77;5.13)	3.12 (2.59;3.69)
4+	Drug related	0.69 (0.46;0.95)	0.47 (0.28;0.70)
4+	Eating disorders	-	0.06 (0.00;0.14)
4+	Suicide	3.32 (2.79;3.91)	3.18 (2.62;3.76)
4+	Accidents	5.86 (5.19;6.60)	3.40 (2.86;3.94)
4+	Homicide	0.20 (0.06;0.39)	-
4+	Other causes of death	1.91 (1.27;2.56)	2.09 (1.45;2.77)

© 2020 Weye N et al. *JAMA Network Open*.

Estimates based on less than three cases are not shown. All-cause LYLs for cocaine use disorder, amphetamine use disorder, eating disorders (overall), anorexia, bulimia, ADHD and conduct disorders are presented in Table 3.

© 2020 Weye N et al. *JAMA Network Open*.

eReferences

1. Naghavi M, Abajobir AA, Abbafati C, et al. Global, regional, and national age-sex specific mortality for 264 causes of death, 1980–2016: a systematic analysis for the Global Burden of Disease Study 2016. *Lancet*. 2017;390(10100):1151-210.

© 2020 Weye N et al. *JAMA Network Open*.
